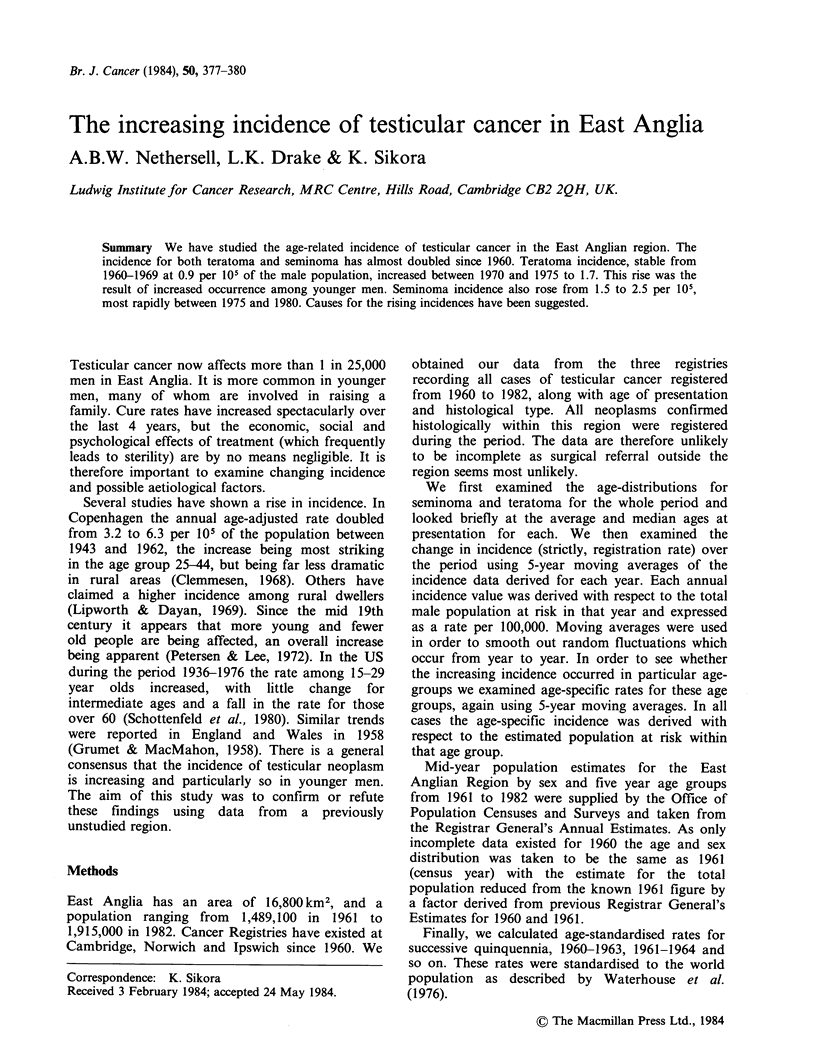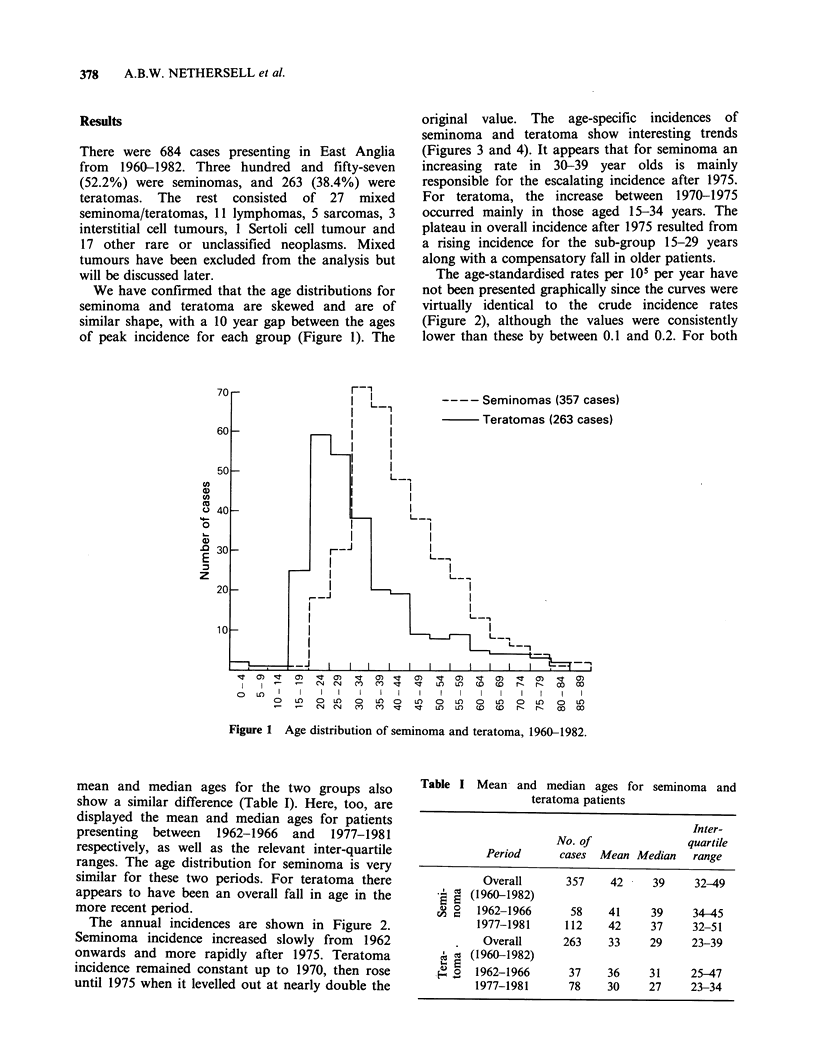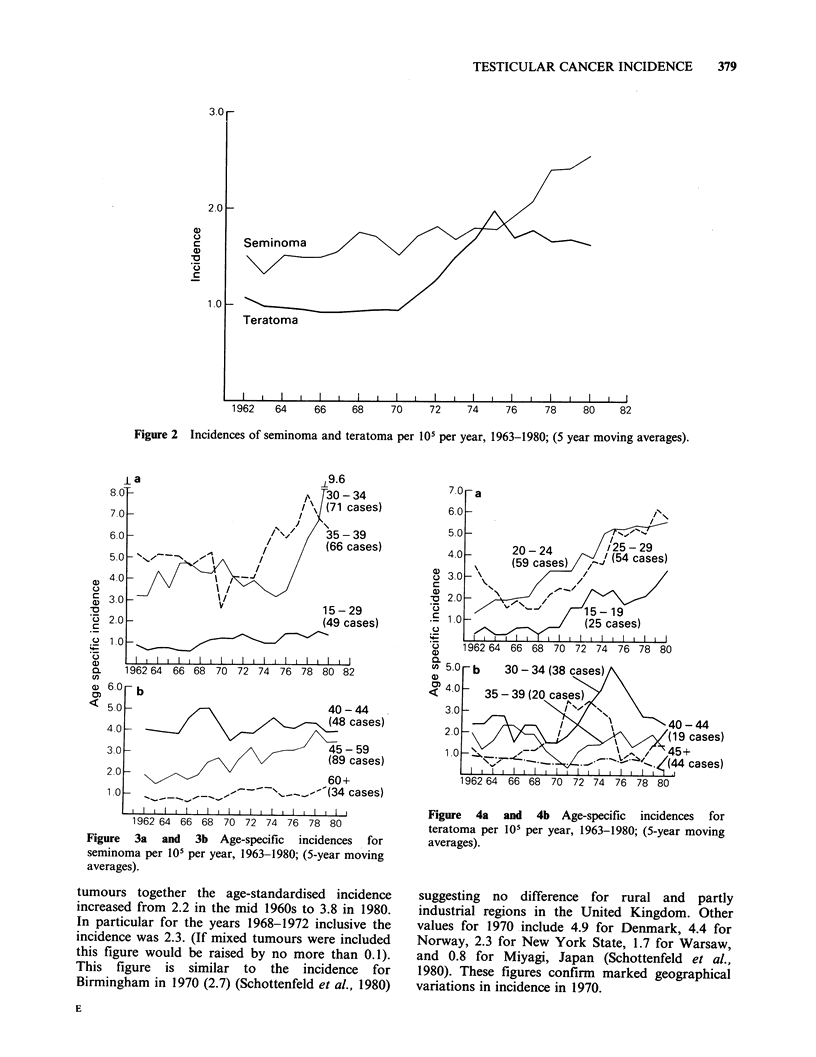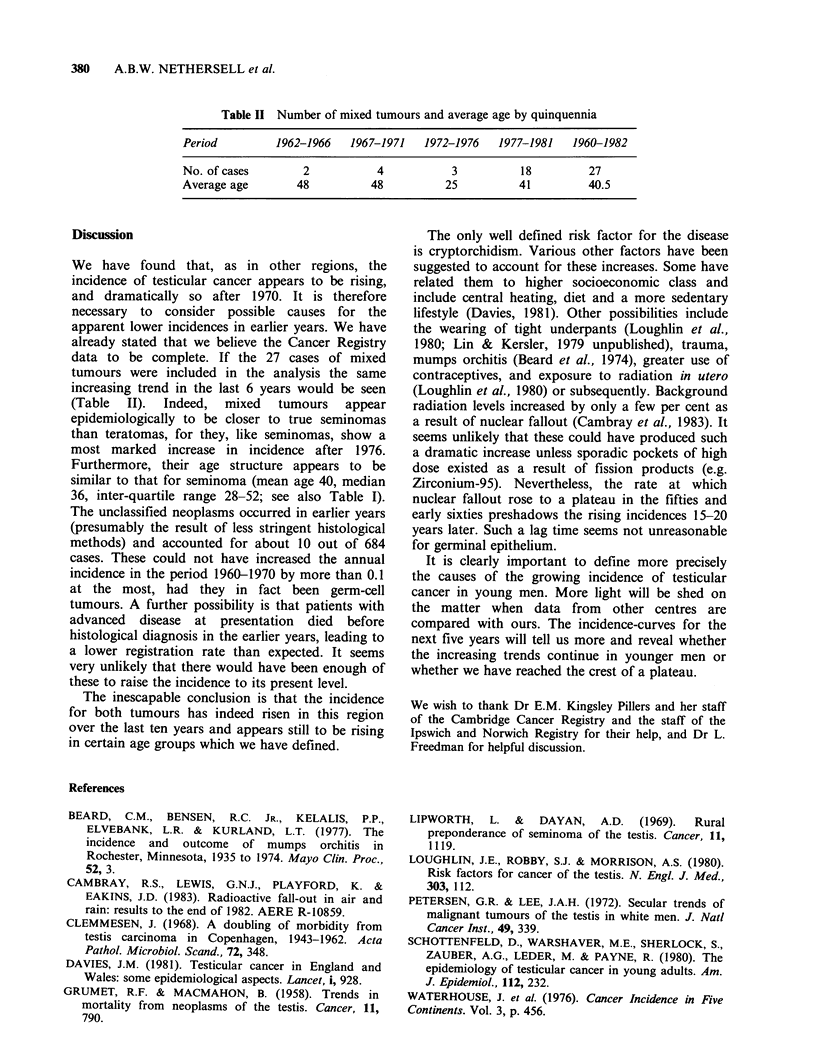# The increasing incidence of testicular cancer in East Anglia.

**DOI:** 10.1038/bjc.1984.186

**Published:** 1984-09

**Authors:** A. B. Nethersell, L. K. Drake, K. Sikora

## Abstract

We have studied the age-related incidence of testicular cancer in the East Anglian region. The incidence for both teratoma and seminoma has almost doubled since 1960. Teratoma incidence, stable from 1960-1969 at 0.9 per 10(5) of the male population, increased between 1970 and 1975 to 1.7. This rise was the result of increased occurrence among younger men. Seminoma incidence also rose from 1.5 to 2.5 per 10(5), most rapidly between 1975 and 1980. Causes for the rising incidences have been suggested.


*Br. J. Cancer* (1984), **50**, 377–380

### The increasing incidence of testicular cancer in East Anglia

A.B.W. Nethersell, L.K. Drake & K. Sikora

*Ludwig Institute for Cancer Research, MRC Centre, Hills Road, Cambridge CB2 2QH, UK.*

**Summary** We have studied the age-related incidence of testicular cancer in the East Anglian region. The incidence for both teratoma and seminoma has almost doubled since 1960. Teratoma incidence, stable from 1960–1969 at 0.9 per $10^5$ of the male population, increased between 1970 and 1975 to 1.7. This rise was the result of increased occurrence among younger men. Seminoma incidence also rose from 1.5 to 2.5 per $10^5$, most rapidly between 1975 and 1980. Causes for the rising incidences have been suggested.

Testicular cancer now affects more than 1 in 25,000 men in East Anglia. It is more common in younger men, many of whom are involved in raising a family. Cure rates have increased spectacularly over the last 4 years, but the economic, social and psychological effects of treatment (which frequently leads to sterility) are by no means negligible. It is therefore important to examine changing incidence and possible aetiological factors.

Several studies have shown a rise in incidence. In Copenhagen the annual age-adjusted rate doubled from 3.2 to 6.3 per $10^5$ of the population between 1943 and 1962, the increase being most striking in the age group 25–44, but being far less dramatic in rural areas (Clemmesen, 1968). Others have claimed a higher incidence among rural dwellers (Lipworth & Dayan, 1969). Since the mid 19th century it appears that more young and fewer old people are being affected, an overall increase being apparent (Petersen & Lee, 1972). In the US during the period 1936–1976 the rate among 15–29 year olds increased, with little change for intermediate ages and a fall in the rate for those over 60 (Schottenfeld *et al.*, 1980). Similar trends were reported in England and Wales in 1958 (Grumet & MacMahon, 1958). There is a general consensus that the incidence of testicular neoplasm is increasing and particularly so in younger men. The aim of this study was to confirm or refute these findings using data from a previously unstudied region.

### Methods

East Anglia has an area of 16,800 km², and a population ranging from 1,489,100 in 1961 to 1,915,000 in 1982. Cancer Registries have existed at Cambridge, Norwich and Ipswich since 1960. We obtained our data from the three registries recording all cases of testicular cancer registered from 1960 to 1982, along with age of presentation and histological type. All neoplasms confirmed histologically within this region were registered during the period. The data are therefore unlikely to be incomplete as surgical referral outside the region seems most unlikely.

We first examined the age-distributions for seminoma and teratoma for the whole period and looked briefly at the average and median ages at presentation for each. We then examined the change in incidence (strictly, registration rate) over the period using 5-year moving averages of the incidence data derived for each year. Each annual incidence value was derived with respect to the total male population at risk in that year and expressed as a rate per 100,000. Moving averages were used in order to smooth out random fluctuations which occur from year to year. In order to see whether the increasing incidence occurred in particular age-groups we examined age-specific rates for these age groups, again using 5-year moving averages. In all cases the age-specific incidence was derived with respect to the estimated population at risk within that age group.

Mid-year population estimates for the East Anglian Region by sex and five year age groups from 1961 to 1982 were supplied by the Office of Population Censuses and Surveys and taken from the Registrar General's Annual Estimates. As only incomplete data existed for 1960 the age and sex distribution was taken to be the same as 1961 (census year) with the estimate for the total population reduced from the known 1961 figure by a factor derived from previous Registrar General's Estimates for 1960 and 1961.

Finally, we calculated age-standardised rates for successive quinquennia, 1960–1963, 1961–1964 and so on. These rates were standardised to the world population as described by Waterhouse *et al.* (1976).

Correspondence: K. Sikora
Received 3 February 1984; accepted 24 May 1984.

© The Macmillan Press Ltd., 1984

### Results

There were 684 cases presenting in East Anglia from 1960–1982. Three hundred and fifty-seven (52.2%) were seminomas, and 263 (38.4%) were teratomas. The rest consisted of 27 mixed seminoma/teratomas, 11 lymphomas, 5 sarcomas, 3 interstitial cell tumours, 1 Sertoli cell tumour and 17 other rare or unclassified neoplasms. Mixed tumours have been excluded from the analysis but will be discussed later.

We have confirmed that the age distributions for seminoma and teratoma are skewed and are of similar shape, with a 10 year gap between the ages of peak incidence for each group (Figure 1). The original value. The age-specific incidences of seminoma and teratoma show interesting trends (Figures 3 and 4). It appears that for seminoma an increasing rate in 30–39 year olds is mainly responsible for the escalating incidence after 1975. For teratoma, the increase between 1970–1975 occurred mainly in those aged 15–34 years. The plateau in overall incidence after 1975 resulted from a rising incidence for the sub-group 15–29 years along with a compensatory fall in older patients.

The age-standardised rates per $10^5$ per year have not been presented graphically since the curves were virtually identical to the crude incidence rates (Figure 2), although the values were consistently lower than these by between 0.1 and 0.2. For both

**Figure 1** Age distribution of seminoma and teratoma, 1960–1982.

mean and median ages for the two groups also show a similar difference (Table I). Here, too, are displayed the mean and median ages for patients presenting between 1962–1966 and 1977–1981 respectively, as well as the relevant inter-quartile ranges. The age distribution for seminoma is very similar for these two periods. For teratoma there appears to have been an overall fall in age in the more recent period.

The annual incidences are shown in Figure 2. Seminoma incidence increased slowly from 1962 onwards and more rapidly after 1975. Teratoma incidence remained constant up to 1970, then rose until 1975 when it levelled out at nearly double the

**Table I** Mean and median ages for seminoma and teratoma patients

| | Period | No. of cases | Mean | Median | Inter-quartile range |
|---|---|---|---|---|---|
| Semi-noma | Overall (1960–1982) | 357 | 42 | 39 | 32–49 |
| | 1962–1966 | 58 | 41 | 39 | 34–45 |
| | 1977–1981 | 112 | 42 | 37 | 32-51 |
| Tera-toma | Overall (1960–1982) | 263 | 33 | 29 | 23–39 |
| | 1962–1966 | 37 | 36 | 31 | 25–47 |
| | 1977–1981 | 78 | 30 | 27 | 23–34 |

**Figure 2** Incidences of seminoma and teratoma per $10^5$ per year, 1963–1980; (5 year moving averages).

**Figure 3a and 3b** Age-specific incidences for seminoma per $10^5$ per year, 1963–1980; (5-year moving averages).

**Figure 4a and 4b** Age-specific incidences for teratoma per $10^5$ per year, 1963–1980; (5-year moving averages).

tumours together the age-standardised incidence increased from 2.2 in the mid 1960s to 3.8 in 1980. In particular for the years 1968–1972 inclusive the incidence was 2.3. (If mixed tumours were included this figure would be raised by no more than 0.1). This figure is similar to the incidence for Birmingham in 1970 (2.7) (Schottenfeld *et al.*, 1980) suggesting no difference for rural and partly industrial regions in the United Kingdom. Other values for 1970 include 4.9 for Denmark, 4.4 for Norway, 2.3 for New York State, 1.7 for Warsaw, and 0.8 for Miyagi, Japan (Schottenfeld *et al.*, 1980). These figures confirm marked geographical variations in incidence in 1970.

**Table II** Number of mixed tumours and average age by quinquennia

| *Period* | *1962–1966* | *1967–1971* | *1972–1976* | *1977–1981* | *1960–1982* |
|---|---|---|---|---|---|
| No. of cases | 2 | 4 | 3 | 18 | 27 |
| Average age | 48 | 48 | 25 | 41 | 40.5 |

### Discussion

We have found that, as in other regions, the incidence of testicular cancer appears to be rising, and dramatically so after 1970. It is therefore necessary to consider possible causes for the apparent lower incidences in earlier years. We have already stated that we believe the Cancer Registry data to be complete. If the 27 cases of mixed tumours were included in the analysis the same increasing trend in the last 6 years would be seen (Table II). Indeed, mixed tumours appear epidemiologically to be closer to true seminomas than teratomas, for they, like seminomas, show a most marked increase in incidence after 1976. Furthermore, their age structure appears to be similar to that for seminoma (mean age 40, median 36, inter-quartile range 28–52; see also Table I). The unclassified neoplasms occurred in earlier years (presumably the result of less stringent histological methods) and accounted for about 10 out of 684 cases. These could not have increased the annual incidence in the period 1960–1970 by more than 0.1 at the most, had they in fact been germ-cell tumours. A further possibility is that patients with advanced disease at presentation died before histological diagnosis in the earlier years, leading to a lower registration rate than expected. It seems very unlikely that there would have been enough of these to raise the incidence to its present level.

The inescapable conclusion is that the incidence for both tumours has indeed risen in this region over the last ten years and appears still to be rising in certain age groups which we have defined.

The only well defined risk factor for the disease is cryptorchidism. Various other factors have been suggested to account for these increases. Some have related them to higher socioeconomic class and include central heating, diet and a more sedentary lifestyle (Davies, 1981). Other possibilities include the wearing of tight underpants (Loughlin *et al.*, 1980; Lin & Kersler, 1979 unpublished), trauma, mumps orchitis (Beard *et al.*, 1974), greater use of contraceptives, and exposure to radiation *in utero* (Loughlin *et al.*, 1980) or subsequently. Background radiation levels increased by only a few per cent as a result of nuclear fallout (Cambray *et al.*, 1983). It seems unlikely that these could have produced such a dramatic increase unless sporadic pockets of high dose existed as a result of fission products (e.g. Zirconium-95). Nevertheless, the rate at which nuclear fallout rose to a plateau in the fifties and early sixties preshadows the rising incidences 15–20 years later. Such a lag time seems not unreasonable for germinal epithelium.

It is clearly important to define more precisely the causes of the growing incidence of testicular cancer in young men. More light will be shed on the matter when data from other centres are compared with ours. The incidence-curves for the next five years will tell us more and reveal whether the increasing trends continue in younger men or whether we have reached the crest of a plateau.

We wish to thank Dr E.M. Kingsley Pillers and her staff of the Cambridge Cancer Registry and the staff of the Ipswich and Norwich Registry for their help, and Dr L. Freedman for helpful discussion.

### References

BEARD, C.M., BENSEN, R.C. JR., KELALIS, P.P., ELVEBANK, L.R. & KURLAND, L.T. (1977). The incidence and outcome of mumps orchitis in Rochester, Minnesota, 1935 to 1974. *Mayo Clin. Proc.*, **52**, 3.

CAMBRAY, R.S., LEWIS, G.N.J., PLAYFORD, K. & EAKINS, J.D. (1983). Radioactive fall-out in air and rain: results to the end of 1982. AERE R-10859.

CLEMMESEN, J. (1968). A doubling of morbidity from testis carcinoma in Copenhagen, 1943–1962. *Acta Pathol. Microbiol. Scand.*, **72**, 348.

DAVIES, J.M. (1981). Testicular cancer in England and Wales: some epidemiological aspects. *Lancet*, **i**, 928.

GRUMET, R.F. & MACMAHON, B. (1958). Trends in mortality from neoplasms of the testis. *Cancer*, **11**, 790.

LIPWORTH, L. & DAYAN, A.D. (1969). Rural preponderance of seminoma of the testis. *Cancer*, **11**, 1119.

LOUGHLIN, J.E., ROBBY, S.J. & MORRISON, A.S. (1980). Risk factors for cancer of the testis. *N. Engl. J. Med.*, **303**, 112.

PETERSEN, G.R. & LEE, J.A.H. (1972). Secular trends of malignant tumours of the testis in white men. *J. Natl Cancer Inst.*, **49**, 339.

SCHOTTENFELD, D., WARSHAVER, M.E., SHERLOCK, S., ZAUBER, A.G., LEDER, M. & PAYNE, R. (1980). The epidemiology of testicular cancer in young adults. *Am. J. Epidemiol.*, **112**, 232.

WATERHOUSE, J. *et al.* (1976). *Cancer Incidence in Five Continents*. Vol. 3, p. 456.